# Exosomes released from macrophages infected with *Talaromyces marneffei* activate the innate immune responses and decrease the replication

**DOI:** 10.1002/iid3.881

**Published:** 2023-06-14

**Authors:** Guangquan Ji, Shan Feng, Hong Ren, Wenhao Chen, Renqiong Chen

**Affiliations:** ^1^ Department of Technology Lianyungang Clinical College of Nanjing Medical University Lianyungang China; ^2^ Department of Technology The First Affiliated Hospital of Kangda College of Nanjing Medical University Lianyungang China; ^3^ Department of Dermatology The First Affiliated Hospital of Kangda College of Nanjing Medical University Lianyungang China; ^4^ Department of Dermatology Affiliated Lianyungang Hospital of Xuzhou Medical University Lianyungang China

**Keywords:** ERK1/2, exosomes, macrophages, *Talaromyces marneffe*i

## Abstract

**Introduction:**

Recent studies have demonstrated that exosomes play roles in pathogenesis and in the treatment of various diseases. We explored the influence of exosomes released from *Talaromyces marneffei* (*T. marneffei*)‐infected macrophages on human macrophages to determine whether they play a role in the pathogenesis of *T. marneffei* infection.

**Methods:**

Exosomes derived from macrophages infected with *T. marneffei* were extracted and characterized by transmission electron microscopy and western blot. Moreover, we examined exosomes that modulated IL‐10 and TNF‐α secretion and activation of p42 and p44 extracellular signal‐regulated kinase 1 and 2 (ERK1/2) and activation of autophagy.

**Results:**

We found that exosomes promoted activation of ERK1/2 and autophagy, IL‐10 and TNF‐α secretion in human macrophages. Further, exosomes decreased the multiplication of *T. marneffei* in *T. marneffei*‐infected human macrophages. Interestingly, exosomes isolated from *T. marneffei*‐infected but not from uninfected macrophages can stimulate innate immune responses in resting macrophages.

**Conclusion:**

Our studies are the first to demonstrate that exosomes isolated from *T. marneffei*‐infected macrophages can modulate the immune system to control inflammation, and we hypothesize that exosomes play significant roles in activation of ERK1/2 and autophagy, the replication of *T. marneffei* and cytokine production during *T. marneffei* infection.

## Graphical abstract

Our studies are the first to demonstrate that exosomes isolated from *T. marneffei*‐infected macrophages can induce a pro‐inflammatory response, and we hypothesize that exosomes play significant roles in activation of ERK1/2 and autophagy, the replication of *T. marneffei* and cytokine release during *T. marneffei* infection.

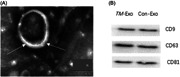

## 1 INTRODUCTION

*Talaromyces marneffei*, formerly named *Penicillium marneffei* is a significant emerging dimorphic fungus that can cause a severe systemic mycosis in humans across a narrow band of tropical South and Southeast Asia.^1^, ^2^, ^3^, ^4^, ^5^

Recent studies have demonstrated that macrophages are the initial immune cells necessary for preventing and controlling infection with *T. marneffei*.^6^, ^7^, ^8^, ^9^, ^10^, ^11^ Further, previous study found that tumor necrosis factor‐a (TNF‐a) decreased the replication of *T. marneffei* in macrophages.^8^ In addition, IL‐10 as a significant regulator of myeloid cells inhibits macrophages to produce cytokines.^12^

ERK1/2 as a member of mitogen‐activated protein kinases (MAPKs) transduces the signals of various stimuli including mitogens, growth factors, and cytokines from the cell surface to the nucleus and regulate cytokine production.^13^ ERK1/2 activation may limit the bacterial and fungal replication and plays important roles in many pathogens such as *T. marneffei*, *Mycobacterium avium*, *Yersinia enterocolitica*, and *Candida albican*s.^11^, ^13^, ^14^, ^15^ Besides, autophagy as a self‐degradative process was essential for the clearance of intracellular pathogens such as *Shigella flexneri*, *Streptococcus pyogenes*, *Mycobacterium tuberculosis* (*M. tuberculosis*) and *T. marneffei*.^16^, ^17^, ^18^, ^19^

Exosomes are small vesicles derived from many eukaryotic cells. Exosomes can be obtained from B cells, macrophages, dendritic cells, and natural killer cells, which are enriched in proteins of the tetraspanin family including CD63 and CD81.^20^ Exosomes released from macrophages infected with *M. tuberculosis* promote both innate and acquired immune responses in vitro.^21^, ^22^

Although the modulatory effects of exosomes released from *M. tuberculosis*‐infected macrophages has been reviewed^23^ and indicate that they can stimulate production of inflammatory mediators, little is known for the role of exosomes in ERK1/2 activation and autophagy, cytokine expression, and replication of *T. marneffei* in *T. marneffei*‐infected macrophages. In this research, the exosomes derived from *T. marneffei*‐infected macrophages were characterized and the effects of the exosomes on human macrophages in vitro were also explored.

## 2 MATERIALS AND METHODS

### 2.1 Reagents

Phospho‐ERK1/2, β‐actin, and ERK1/2 antibodies came from Cell Signaling Technology. Goat anti‐rabbit IgG and goat anti‐mouse IgG were obtained from Santa Cruz Biotechnology. LC‐3 antibody was purchased from Sigma Chemical Co.

### 2.2 *T. marneffei* fungal strain

*T. marneffei* strain, named B33w was purchased from Chinese Medicine Mycology Database. It grew on potato dextrose agar (PDA) at 25°C for 14 days. *T. marneffei* conidia were collected and washed with phosphate‐buffered saline before each experiment.

### 2.3 Isolation and culture of human macrophages

Peripheral blood mononuclear cells were isolated according to standard procedures described in our previous study by Ficoll‐Paque. The cells were incubated for 1 h at 37°C, and nonadherent cells were removed. Macrophages were obtained by culturing the monocytes for 7–8 days.

### 2.4 In vitro infection

Macrophages were infected with *T. marneffei* conidia at a multiplicity of infection of 5:1. Further, macrophages were treated with or without exosomes.

### 2.5 Exosomes isolation

Macrophages were stimulated with *T. marneffei* conidia for 48 h and then supernatants were centrifuged at 3000*g* for 15 min to remove cells and cell debris. The ExoQuick Exosome Precipitation Solution (System Biosciences [SBI]) was added to supernatants according to the procedure. Exosomes were quantified using the Micro BCA Protein Assay and stored at −80°C for following experiments.

### 2.6 Transmission electron microscopy

We detected exosomes by transmission electron microscopy. The prepared exosome eluate was stained with 3% phosphotungstic acid solution for 3–5 min and viewed on a JEOL‐Jem‐1200EX (JEOL) transmission electron microscope.

### 2.7 Western blot analysis

Macrophages were treated with 25 μg/mL exosomes within 120 min. For western blot analysis, proteins from cell lysates, as determined by the Micro BCA Protein Assay, were loaded on 12% SDS‐PAGE gels, electrophoresed, and transferred onto PVDF membrane. The membranes were probed for the primary antibody (anti‐CD63, anti‐CD9, anti‐CD81 anti‐ERK1/2, anti‐phospho‐ERK1/2, anti‐LC3, or anti‐β‐actin). Immunodetected bands were developed using chemiluminescent substrate.

### 2.8 Cytokine assays

Macrophages were treated with or without 12.5, 25, and 50 μg/mL exosomes for 24 h. Supernatants were harvested and kept at −80°C until analysis and were measured for TNF‐α and IL‐10 levels by enzyme‐linked immunosorbent assay (ELISA) using a commercial kit (R&D Systems).

### 2.9 Determination of colony forming units (CFUs)

To study the impact of exosomes on the multiplication of *T. marneffei*, macrophages were infected with *T. marneffei* containing with or without 25 and 50 μg/mL exosomes for 24 h. Lysates were serially diluted and plated on Sabouraud dextrose agar (SDA) at 37°C in triplicate. CFUs were counted after 3 days.

### 2.10 Statistical analysis

Data are shown as mean ± standard deviation by means of a Student's *t* test or one‐way analysis of variance using GraphPad Prism 5.0 (GraphPad Software, Inc.). Values of *p* < .05 were defined as statistically significant. All experiments were repeated at least three times.

## 3 RESULTS

### 3.1 Identification of exosomes derived from macrophages

**Figure 1:**
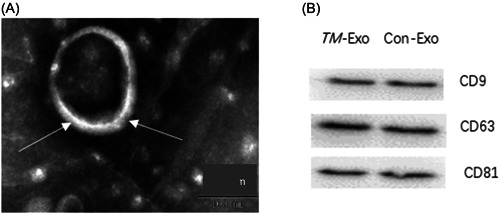
Identification of exosomes derived from *Talaromyces marneffei‐infected* macrophages. (A) The morphology of exosomes, as shown by transmission electron microscopy. (B) Expression levels of CD9, CD63, and CD81 in macrophage‐derived exosomes stimulated by *T*. *marneffei* (*TM*‐Exo), as shown by western blot analysis. Con‐Exo, exosomes derived from naïve macrophages.

TEM images showed that exosomes exhibited vesicle morphology and ranged in size from 30 to 100 nm (Figure 1A). The results of western blot analysis confirmed the expression of CD9, CD63, and CD81 in exosomes (Figure 1B).

### 3.2 Exosomes induced TNF‐α and IL‐10 production in human macrophages

**Figure 2:**
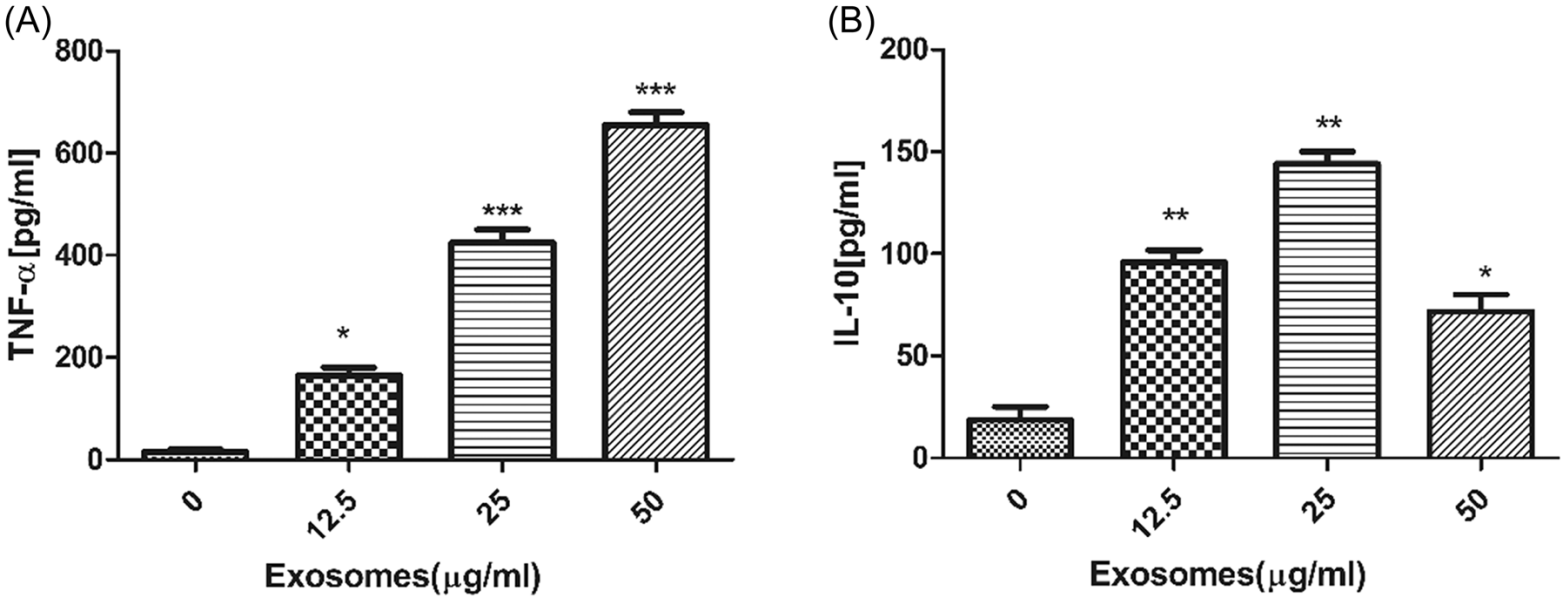
Exosomes induced TNF‐α and IL‐10 production in human macrophages. Macrophages were treated with different doses of exosomes. The levels of TNF‐α (A) and IL‐10 (B) were measured after the times indicated by ELISA. Data are expressed as the mean ± standard deviation by a one‐way analysis of variance. All experiments were repeated at least three times. TNF‐α, tumor necrosis factor‐a. *p < .05, **p < .01, ***p < .001.

To examine whether exosomes could promote macrophages activation, the production of TNF‐α (Figure 2A) and IL‐10 (Figure 2B) from human macrophages treated with exosomes was detected. The results indicated that TNF‐α and IL‐10 secretion was increased when compared with controls.

### 3.3 Exosomes induced phosphorylation of ERK1/2 in human macrophages

**Figure 3:**
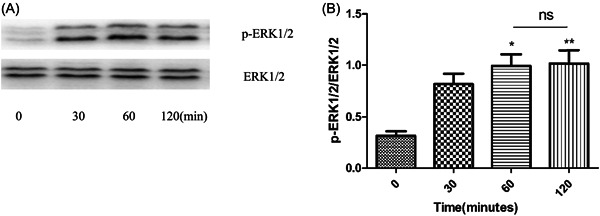
Exosomes induced phosphorylation of ERK1/2 in human macrophages. Human macrophages were stimulated with or without 25 μg/mL exosomes for the time interval indicated. The phosphorylation of ERK1/2 in macrophages were measured by Western blot (A) and quantification of ERK1/2 (B) was performed by densitometric analysis. Membranes were stripped and reprobed for total ERK 1/2. Data are expressed as the mean ± standard deviation by a one‐way analysis of variance. All experiments were repeated at least three times. ns, not significant. *p < .05, **p < .01.

To study the influence of exosomes on ERK1/2, the phosphorylation of ERK1*/*2 in human macrophages treated with exosomes was therefore evaluated (Figure 3A,B). Exosomes induced strong phosphorylation of ERK1/2 within 120 min. The peak of phosphorylation of ERK1/2 occurred within 60–120 min.

### 3.4 Autophagy induction increased in human macrophages upon the exosomes

**Figure 4:**
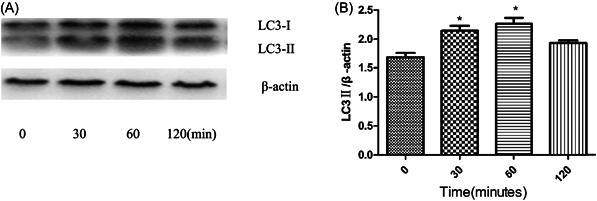
Autophagy induction increased in human macrophages upon the exosomes. Human macrophages were incubated with or without 25 μg/mL exosomes for the time interval indicated. (A) The levels of LC3B‐I, LC3B‐II protein in human macrophages were examined by Western blot and quantification of LC3B‐II (B) was performed by densitometric analysis. Data are expressed as the mean ± standard deviation by a one‐way analysis of variance. All experiments were repeated at least three times. *p < .05.

To investigate increased autophagy stimulated by exosomes, macrophages were incubated with or without exosomes for the time interval indicated. Along with the higher conversion of LC3B‐I to LC3B‐II in exosomes‐stimulated macrophages also increased, suggesting that exosomes promote autophagic flux in macrophages. Exosomes induced activation of autophagy within 120 min. The peak of autophagic flux occurred within 30–60 min (Figure 4A,B).

### 3.5 Effect of exosomes on intracellular replication of *T. marneffei* in human macrophages

**Figure 5:**
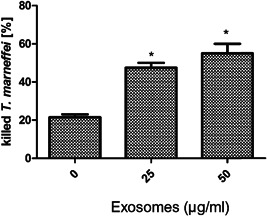
Effect of exosomes on intracellular replication of *Talaromyces marneffei* in human macrophages. Macrophages were pretreated with or without exosomes for 1 h, and then infected with *T. marneffei* for 24 h. The cells were lysed and plated onto SDA in serial dilutions for CFU assay. Data are expressed as the mean ± standard deviation by a one‐way analysis of variance. All experiments were repeated at least three times. *p < .05.

As shown above, *T. marneffei* induced the release of exosomes within macrophages. To examine the effect of exosomes on the replication of *T. marneffei*, macrophages were pretreated with different concentrations of exosomes for 1 h, and then cultured with *T. marneffei* for 24 h. Exosomes decreased the replication of *T. marneffei* in human macrophages (Figure 5).

## 4 DISCUSSION

Exosomes are derived from cell endosomal membrane system and play a role in regulating immune responses. Previous studies have shown that exosomes released from *M. tuberculosis*‐infected cells can induce a pro‐inflammatory response when exposed to macrophages.^24^ Further, exosomes were verified containing many marker proteins, such as heat shock proteins, CD63, CD9, CD81, and CD82. There were several studies on virulence factors of *T. marneffei* such as heat shock proteins, antioxidant enzymes, MP1p, and nutritional metabolism‐related enzymes.^25^, ^26^, ^27^, ^28^ However, the pathogenesis of *T. marneffei* has not been fully elucidated. Also, a recent study found that some proven or putative virulence factors, including heat shock proteins, MP1p, and peroxidase, were also identified in the proteome of *T. marneffei*‐derived extracellular vesicles.^29^ In the study, we found that exosomes were derived from *T. marneffei*‐infected macrophages and expressed CD63, CD9, and CD81. Importantly, exosomes promoted activation of autophagy and ERK1/2, IL‐10, and TNF‐α secretion in human macrophages. Moreover, exosomes reduced the replication of *T. marneffei* in *T. marneffei*‐infected human macrophages.

In our previous study, we have found that *T. marneffei* causes a significant increase in the secretion of both IL‐10 and TNF‐a by macrophages.^11^ Interestingly, we found similar results, particularly regarding TNF‐a and IL‐10, when macrophages were treated with exosomes from *T. marneffei*‐infected macrophages. This is similar to the results that exosomes released from infected cells contain *M. avium* glycopeptidolipids and are pro‐inflammatory.^30^

ERK1/2 is activated by a variety of infections and takes part in the induction of innate immunity. Previous studies showed that both *C. albicans* and *Saccharomyces cerevisiae* blastoconidia in J774 cells activated the ERK1/2 pathway.^31^ Also, *T. marneffei* induced ERK1/2 phosphorylation in human macrophages.^11^ Further, the ERK signaling pathway has been reported to be the major positive regulator of autophagy.^15^ In addition, inhibition of ERK1/2 pathway increases the replication of *T. marneffei*. Numerous studies reported that exosomes isolated from cells infected with various intracellular pathogens contain microbial components and could promote antigen presentation and macrophage activation.^32^, ^33^ To determine the role of exosomes in activation to macrophages, we examined the impact of exosomes on macrophages. In this study, we found that exosomes induced phosphorylation of ERK1/2. Besides, exosomes could inhibit the replication of *T. marneffei*. These results showed that exosomes, causing of ERK1/2 activation and translocation to nucleus, which might be a significant regulator of host‐pathogen interactions.

As we know, autophagy is a nonapoptotic form of programmed cell death and it has played an important role in cell immune responses. In this study, we explored the impact of exosomes on the formation of autophagosomes in macrophages. LC3 is the only known mammalian protein that associates with the autophagosome membrane. Upon activation of autophagy, LC3 transformed from LC3I into LC3II.^34^, ^35^ In this research, exosomes not only induced the LC3II protein, but also importantly increased the LC3II/β‐actin ratio. Because the amount of LC3II is connected with the number of autophagosomes, it may be as a good indicator of autophagosome formation.^36^

**Figure 6:**
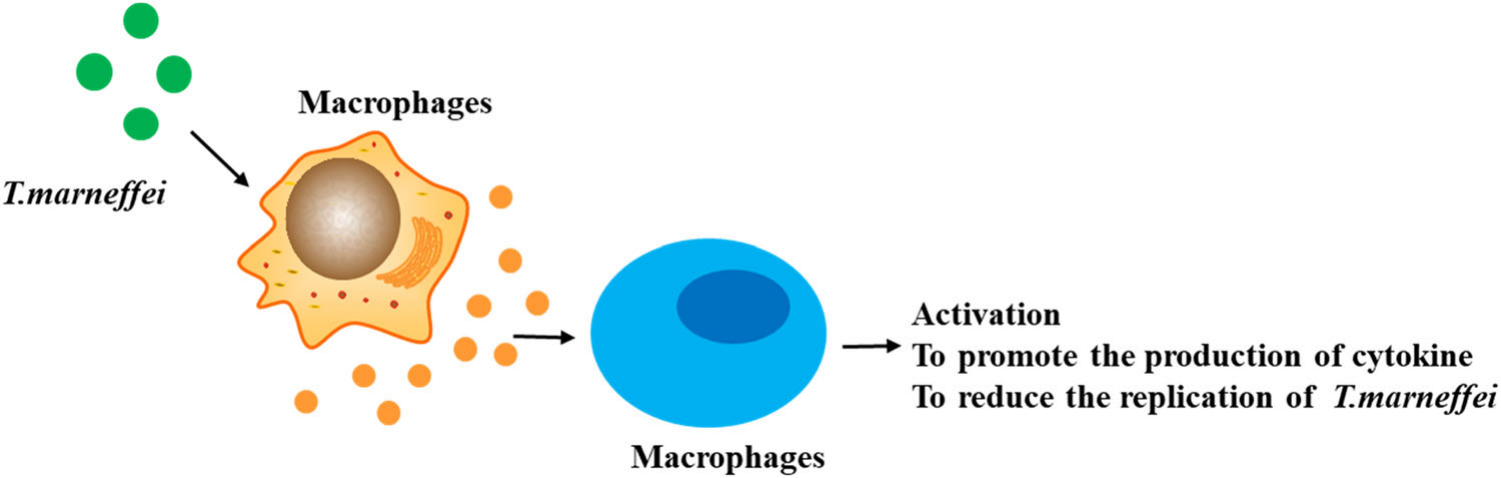
A model that shows the effects of exosomes from *Talaromyces marneffei‐*infected macrophages on human macrophages. Exosomes released from *T. marneffei*‐infected macrophages can promote macrophage activation, release of cytokines, and killing *T. marneffei*.

In general, our study indicated that exosomes released from *T. marneffei*‐infected macrophages can promote macrophage activation, release of cytokines, and killing *T. marneffei* (Figure 6). Our results suggest that exosomes play vital roles in immune response. The study could provide a novel mediator for host‐pathogen interactions and the development of novel approaches to defend this pathogen. Further studies are needed to test whether virulence factors were from *T. marneffei*‐derived exosomes.

## AUTHOR CONTRIBUTIONS

**Shan Feng**: Conceptualization; methodology. **Hong Ren**: Conceptualization; resources. **Wenhao Cheng**: Methodology; software. **Guangquan Ji**: Investigation; methodology; writing—original draft. **Renqiong Chen**: writing—original draft; software.

## CONFLICT OF INTEREST STATEMENT

The authors declare no conflict of interest.
